# Semisupervised Deep Learning Techniques for Predicting Acute Respiratory Distress Syndrome From Time-Series Clinical Data: Model Development and Validation Study

**DOI:** 10.2196/28028

**Published:** 2021-09-14

**Authors:** Carson Lam, Chak Foon Tso, Abigail Green-Saxena, Emily Pellegrini, Zohora Iqbal, Daniel Evans, Jana Hoffman, Jacob Calvert, Qingqing Mao, Ritankar Das

**Affiliations:** 1 Dascena, Inc Houston, TX United States

**Keywords:** acute respiratory distress syndrome, COVID-19, semisupervised learning, deep learning, machine learning, algorithm, prediction, decision support

## Abstract

**Background:**

A high number of patients who are hospitalized with COVID-19 develop acute respiratory distress syndrome (ARDS).

**Objective:**

In response to the need for clinical decision support tools to help manage the next pandemic during the early stages (ie, when limited labeled data are present), we developed machine learning algorithms that use semisupervised learning (SSL) techniques to predict ARDS development in general and COVID-19 populations based on limited labeled data.

**Methods:**

SSL techniques were applied to 29,127 encounters with patients who were admitted to 7 US hospitals from May 1, 2019, to May 1, 2021. A recurrent neural network that used a time series of electronic health record data was applied to data that were collected when a patient’s peripheral oxygen saturation level fell below the normal range (<97%) to predict the subsequent development of ARDS during the remaining duration of patients’ hospital stay. Model performance was assessed with the area under the receiver operating characteristic curve and area under the precision recall curve of an external hold-out test set.

**Results:**

For the whole data set, the median time between the first peripheral oxygen saturation measurement of <97% and subsequent respiratory failure was 21 hours. The area under the receiver operating characteristic curve for predicting subsequent ARDS development was 0.73 when the model was trained on a labeled data set of 6930 patients, 0.78 when the model was trained on the labeled data set that had been augmented with the unlabeled data set of 16,173 patients by using SSL techniques, and 0.84 when the model was trained on the entire training set of 23,103 labeled patients.

**Conclusions:**

In the context of using time-series inpatient data and a careful model training design, unlabeled data can be used to improve the performance of machine learning models when labeled data for predicting ARDS development are scarce or expensive.

## Introduction

Acute respiratory distress syndrome (ARDS) is a broadly defined clinical syndrome associated with significant morbidity and mortality [1,2]. ARDS has been critically misdiagnosed and underdiagnosed despite the high ARDS-associated mortality rates and high rates of related hospital resource use [2-4]. Confidence in ARDS diagnosis varies due to the heterogeneity in disease presentation [5] as well as the heterogeneity in the disease’s definition [6,7]. The identification of ARDS across clinical settings remains subjective [8], and it can be difficult to diagnose the syndrome in patients with underlying conditions that have similar symptom presentations, such as pneumonia [9].

Early intervention is critical to improving patient outcomes, yet there remains a need for clinical decision support tools that can accurately predict ARDS development prior to onset. Per the current Berlin definition of ARDS [10], a radiology report is required to diagnose ARDS. However, rapid radiology reports are often unavailable due to a lack of access to equipment or the lack of the consideration of ARDS by clinicians [11]. The variability in ARDS presentation also makes it challenging to predict ARDS development by using standard machine learning methods, which typically require large amounts of confidently labeled data for supervised learning [12]. Semisupervised learning (SSL) paradigms have been applied to the tasks of biological data [13] classification and microRNA [14] classification and to many similar classification tasks in the domain of biotechnology [15-17] to address the dual issues of poor label quality and limited data quantity. In the context of early ARDS prediction, SSL is useful because it allows for the implicit specification of a useful gold standard. An SSL model schema that integrates information from many clinical features (including radiology reports) during training but only requires a small set of readily available clinical features to make predictions based on test data may, in practice, be crucial to improving early ARDS prediction. The aim of this study was to provide a proof of concept that SSL may be useful for predicting ARDS onset.

## Methods

### Data Sets

**Figure 1:**
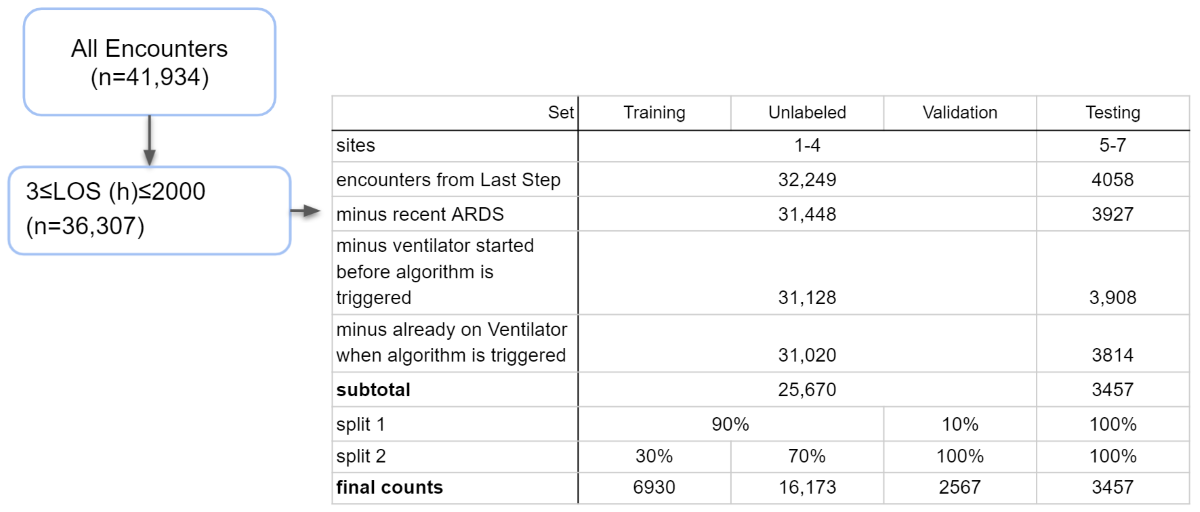
Sample size allocation in the data set. ARDS: acute respiratory distress syndrome; LOS: length of stay.

Data from 7 hospital systems were used in this study, including data from patients who were monitored in emergency department, inpatient ward, and intensive care unit settings. All data were collected passively and deidentified in compliance with the Health Insurance Portability and Accountability Act. Patients with a length of hospital stay of at least 3 hours were included, and positive encounters were defined by the gold standard described in the *Gold-Standard Labels* section. The data set was divided into hold-out test sets, training sets, validation sets, and unlabeled sets, as shown in Figure 1. In order to set aside an external hold-out test set, patients from 3 of the 7 hospital systems were considered to be a part of the test set, and there was no overlap between the patients in this test set and the patients from the remaining 4 hospital systems that were used for the validation, training, and unlabeled sets. Of the 25,670 patients from the nontest set, 2567 (10%) were set aside for the validation set. Of the remaining 23,103 nonvalidation, nontest patients, 6930 (30%) were set aside for the labeled data set, and 16,173 (70%) were set aside for the unlabeled data set. The true label of the unlabeled data set, by definition, was never revealed during the SSL process.

### Gold-Standard Labels

A patient was defined as developing ARDS if a new diagnosis of ARDS based on *International Classification of Diseases* (ICD) codes appeared in the patient’s chart and if we could verify (ie, by using the physiologic time-series data) that the patient experienced respiratory failure. A new code was defined as a code that appeared after admission and was not present during the 1000 hours leading up to admission. In total, 7 outcomes were labeled for each patient, as follows:

1. A clinical diagnosis of ARDS was determined by using ICD codes. The ARDS ICD codes used were J80, J96.0, J96.2, J96.9, and 518.81.
2. Respiratory failure was defined according to the accepted criteria for respiratory failure (a peripheral oxygen saturation [SpO_2_] level of <92% or a partial pressure of oxygen [PaO_2_]/fraction of inspired oxygen [FiO_2_] ratio of <300) [18]. These were approximately corresponding points on the oxygen-hemoglobin dissociation curve, and they allowed us to identify the earliest possible time point in which respiratory failure occurred, even when the PaO_2_ level had not been measured. The prediction of ARDS development leading to respiratory failure was the primary task, and the area under the receiver operating characteristic curve (AUROC) and area under the precision recall curve (AUPRC) were computed and reported based on this label. Although they were not the primary focus of this paper, secondary auxiliary outcomes were used as well.
3. A COVID-19 diagnosis was defined as a positive polymerase chain reaction test for new COVID-19 ICD codes—U07.1, B97.21, B97.29, J12.81, and B34.2.
4. Acute kidney injury was defined by using the following ICD codes: N17, N19, and R34.
5. A broad class of thrombosis was defined by using the following ICD codes: I12, I26, I63, I67, I74, I80, I81, and I82.
6. Sepsis was defined by using the following ICD codes: A40, A41, R65.2, T81.12, T81.44, O85, and O86.04.
7. Patients were labeled according to whether—after a drop in SpO_2_ (below 97%)—they were eventually placed on mechanical ventilation.

### Onset Time

The time point for which the algorithm prediction was outputted was the first time point when the SpO_2_ level fell below the lower range of normal (SpO_2_<97%). This was referred to as the *prediction time*. The onset time for ARDS-positive encounters was defined as the first time point at which any ARDS-related ICD code was found in a patient’s electronic health record (EHR). The onset time for respiratory failure was the first time point when the SpO_2_ level fell below 92% or the PaO_2_/FiO_2_ ratio fell below 300. To find these time points, our data processing function first analyzed all of the SpO_2_ values that were measured for any given patient; if any measurements were <97%, we saved the date-time entry. After this below-97% measurement was collected, we proceeded to determine if the following two later events occurred:

1. The addition of an ARDS ICD code into the EHR. If found, the date-time entry for this event was saved, and the date-time entry for the below-97% SpO_2_ event was subtracted from that of the subsequent measuring event before converting the time difference to hours and plotting the data in a histogram.
2. The subsequent measuring of an SpO_2_ level of <92% or a PaO_2_/FiO_2_ >ratio of <300. If found, the date-time entry for this event was saved, and the date-time entry for the below-97% SpO_2_ event was subtracted from it before converting the time difference to hours.

### Input Features

ARDS predictions were made by using a defined set of data types or features across all hospitals, regardless of the data availability at a particular hospital. Model input features were chosen based on the efficiency at which the features could be extracted from EHRs, feature availability, and consultation with clinicians. For example, most definitions of ARDS require lung findings to be present in the absence of heart failure [3]. The feature availability for the data set is presented in Figure S1 in Multimedia Appendix 1. The model input features consisted of the following: age, gender, the initiation of antibiotics prior to the prediction time, the initiation of supplemental oxygen prior to the prediction time, a history of heart failure, systolic and diastolic blood pressure, heart rate, temperature, respiratory rate, SpO_2_ level (pulse oximetry), creatinine level, blood urea nitrogen level, bilirubin level, glucose level, the international normalized ratio, white blood cell count, red blood cell count, platelet count, percent neutrophil count, percent lymphocyte count, percent monocyte count, hematocrit level, lactate level, aspartate transaminase level, and alanine transaminase level. Not all features were required for the model to make a prediction of ARDS onset.

### Data Processing

The time-series data were organized as a matrix with rows that represented features and columns that represented update time steps. This method of organizing time-series clinical data was the same method used by Che et al [19]. Each column represented a time step in which an update had occurred for one of the features. For simplicity, the first 6 rows represented the following constant features: age, male gender, female gender, the initiation of antibiotics prior to the prediction time, the initiation of supplemental oxygen prior to the prediction time, and a history of heart failure. Except for age, which was normalized by using the mean and SD of the training set, the remaining constant features were coded as 1 or 0. The time series features each had 2 rows—one row contained missingness masks (ie, measurements that were current for a given time step were coded as 1; otherwise, they were coded as 0), and the other row contained the normalized value of current measurements. Further, a row was used to denote the minutes that had passed since the last time step. This was normalized according to the mean and SD of the duration of time between time steps in the training set. To manage memory usage, we set a limit of 32 time steps prior to the prediction time. For patients with less than 32 time steps prior to the prediction time, we performed zero-padding and represented the resulting values as missing data by using a 0 in the missingness mask row. Details of our missing data processing methodology are presented in Table S1 in Multimedia Appendix 1.

### Machine Learning Models

The recurrent neural network (RNN) was implemented with the PyTorch package (version 1.40) in Python 3.6 [20]. The demographics and time series measurements were organized into a sequence of vectors and normalized before being passed to the RNN component of the model by using a normalization layer, as follows:

n(v) = a ⊙ ([v – μ]/[σ + ε]) + b **(1)**

In equation 1, n(v) is a normalization function that learns the parameters mean (*μ*), SD (*σ*), scaling factor *a*, and translation factor *b* to normalize vector embeddings (*v*). The symbol “⊙” is the Hadamard product (also known as the element-wise product). *ε* was set to 1e^−7^ to prevent division-by-zero errors. For the RNN, a sequence module—a 2-layer gated recurrent unit (GRU) [21] with 64 hidden units—was used. A soft attention module was used to assign scores to each time step in the sequence. The attention score was a learned importance weight for each time step. This weight was converted into a probability distribution and multiplied by each sequence’s deepest hidden activation in the GRU to create a weighted sum of the activations, which is called the *context vector*. We concatenated the context vector to the final GRU embedding and passed this vector to a 2-layer feed-forward neural network to produce an output vector for classification. The output vector's length (7 dimensions) was equal to the number of target labels. The intermediate layer before the output logits was a 64D representation of each patient, which was referred to as the *penultimate embedding*. Similar to the method used by Bahdanau et al [21], the score of the attention neural network was parameterized by a feed-forward neural network, as follows:

score(l, h) = K ⋅ tanh (A ⋅ prelu(B ⋅ n([l, h]))) **(2)**

In equation 2, tanh and prelu denote the hyperbolic tangent function and parameterized rectified linear unit nonlinearity functions, respectively. *l* denotes the last value in the sequence and the deepest hidden activation in the GRU, *h* denotes each and any hidden activation in the deepest layer of the GRU in the sequence, and [*l*, *h*] denotes the concatenation of and into a longer vector (the length of the individual vectors were added together). *K*, *A*, and *B* denote the learned matrix parameters of the neural network. The symbol “⋅” denotes matrix multiplication.

**Figure 2:**
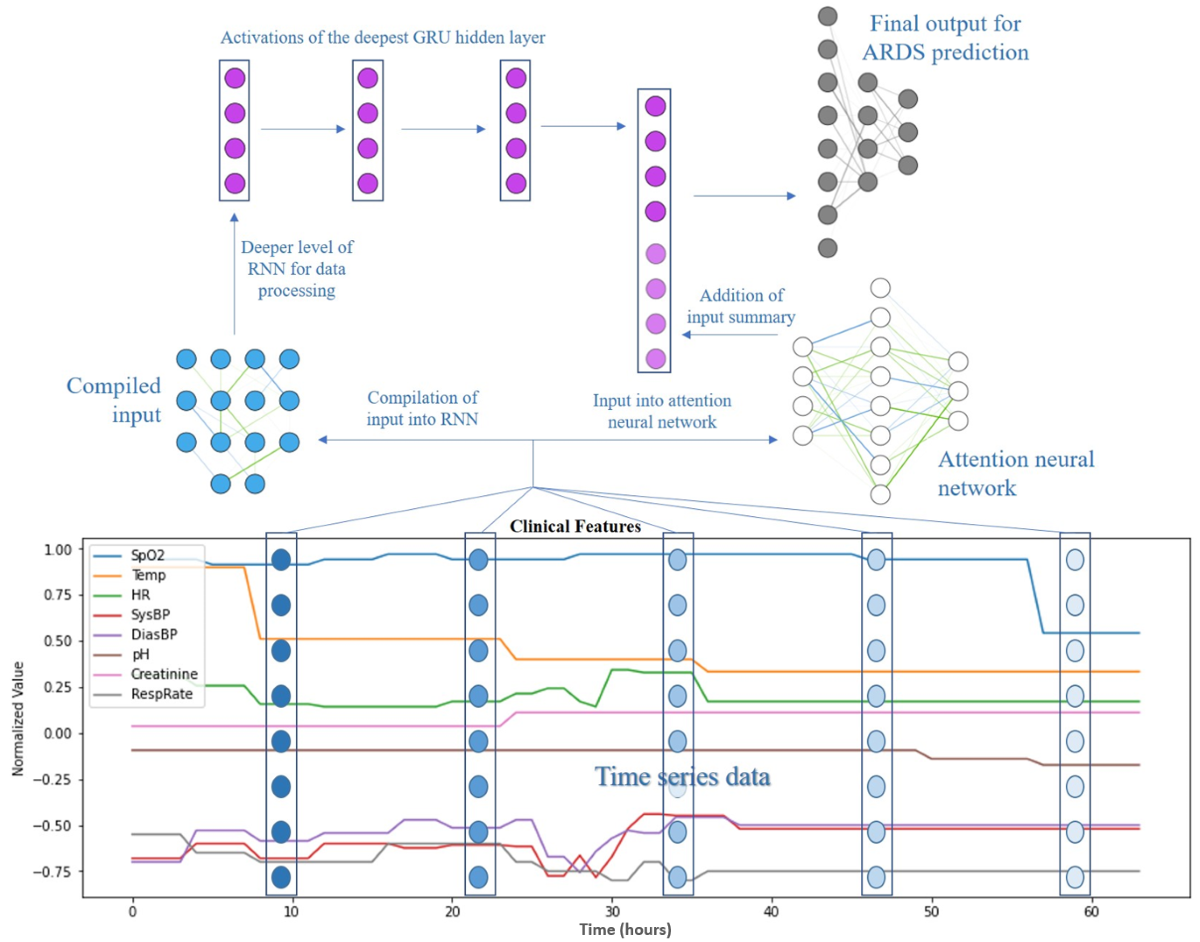
RNN model schema. ARDS: acute respiratory distress syndrome; DiasBP: diastolic blood pressure; GRU: gated recurrent unit; HR: heart rate; RespRate: respiratory rate; RNN: recurrent neural network; SpO_2_: peripheral oxygen saturation; SysBP: systolic blood pressure; Temp: temperature.

The whole GRU-RNN, attention module, and classification module were end-to-end differentiable, which allowed for optimization from input to output. The attention neural network was a mechanism of the RNN that allowed for higher quality learning. Rather than summarizing a time series of vectors, the attention neural network assigned each vector a score according to how important the vector was in terms of allowing the model to make a prediction. As such, the attention network mechanism allowed the RNN to focus on specific parts of the input, thereby improving model performance. The RNN model schema is presented in Figure 2.

Each point in the RNN model schema was representative of a neuron. The neurons received data input from vital signs and laboratory measurements that were recorded in EHRs. At each layer, the RNN combined information from the current and previous time points to update the activations in the deepest hidden layer of the GRU, which, when combined with the importance-weighted average generated by the attention neural network, created a summary of all time-series data—the context vector. The last layer was a feed-forward neural network, which used the activation size of the last deepest hidden state in the GRU combined with the context vector (64+64=128) as input data. With this RNN schema, the model was trained to predict the primary and auxiliary target labels simultaneously and to evaluate a loss function based on all targets.

### Model Training

#### Overview

Our method of SSL was a combination and adaptation of the methodology that was previously developed by Li et al [22] and Xie et al [23]. Rather than performing whole-document and image classification, which were conducted in these prior studies, our models were designed to perform their prediction task by using multivariate time-series data. Our models were tasked with predicting ARDS onset in both the general population and patients with COVID-19.

#### Initial Pseudolabeling

Our methodology builds on our prior work [24]; we simplified the prediction time and inputs for the model. The RNN was first trained on the labeled training set, without making use of the unlabeled set, until convergence occurred in the validation set (keeping the model with the most minimal validation loss). The first RNN was called the *pseudolabeler* or *initial teacher*. The initial teacher was used to predict the probability of future ARDS and auxiliary target development for every patient in the unlabeled set. The mean probability was used as the threshold for the temporary label (the pseudolabel). If the initial teacher assigned a probability that was higher than twice the mean probability for that sample, the sample was considered to be positive and added to the SSL pseudolabeled data set for this cycle of training. If the initial teacher assigned a probability that was below the mean, the sample was considered to be negative and added to the SSL pseudolabeled data set. The remaining samples were not used for this cycle of training because they were considered to be “unconfident.”

#### Semisupervised Relabeling

**Figure 3:**
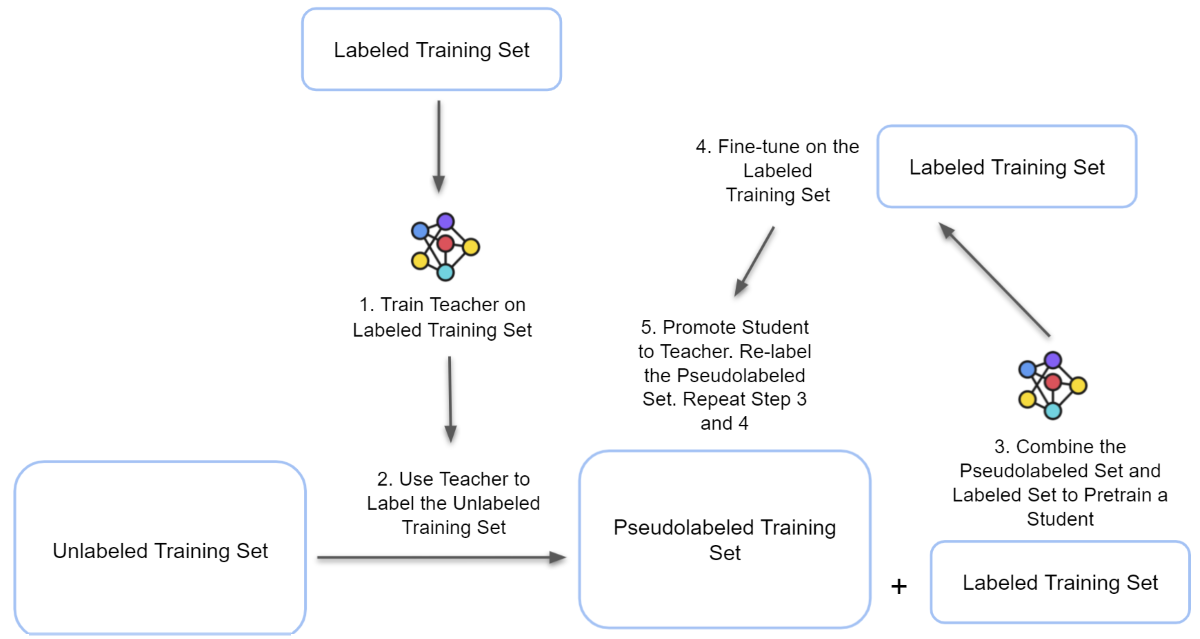
Semisupervised learning schema. The colored network represents the initiation of a new model.

An RNN was used as the semisupervised learner or student machine learning algorithm. For each cycle of SSL, the student machine learning algorithm was trained on the combined labeled and pseudolabeled training set. Afterward, it was fine-tuned on the labeled training set. The student machine learning algorithm then became the teacher for the next cycle of SSL by relabeling the pseudolabeled (unlabeled) training set. The SSL training setup was not meant to perform well on the auxiliary targets; instead, the 6 auxiliary outcomes were used as a multitasking form of regularization for the primary problem. The validation set was used for both hyperparameter selection and the prevention of overfitting only with respect to the ARDS outcome and not with respect to the other outcomes. The pseudolabeling and selection of “confident” labels for the next SSL cycle was performed only with respect to the ARDS outcome and not with respect to the other outcomes. A new RNN was initialized, and the cycle was repeated. Models were trained for 40 epochs, and the model with the best validation set performance was saved (Figure 3).

RNN training was performed by using the Adam optimizer [25] with a decay scheduler to scale down the learning rate (starting from 0.001) by a factor of 0.9 when the multiclass binary cross-entropy loss increased over 2 epochs. A batch size of 2048 was parallelized over 4 Nvidia Tesla M60 (Nvidia Corporation) graphics processing units.

#### Performance Evaluation

Following SSL training, the initial teacher and student models were evaluated for their performance on a hold-out test set based on the AUROC, AUPRC, sensitivity, specificity, positive predictive value, and negative predictive value. The initial teacher performance on the test set defined the baseline performance that SSL was meant to improve upon. In addition to reporting this SSL performance, to define a ceiling for performance, we also compared SSL performance to the performance of a model that was trained on the labeled set and unlabeled set by using the gold-standard labels for both sets instead of the pseudolabels. This model, which was trained on the nonvalidation, nontest patient data, was referred to as the *all data model*. Principal component analysis and t-stochastic neighbor embedding were used to conduct dimensionality reduction and perform a cluster analysis on the RNN’s intermediate representations.

## Results

Demographically, patients with ARDS were similar to patients without ARDS. Except for cardiovascular disease, including heart failure, patients with ARDS had a higher incidence of chronic pulmonary disease, hypertension, diabetes, and obesity (Table 1).

**Figure 4:**
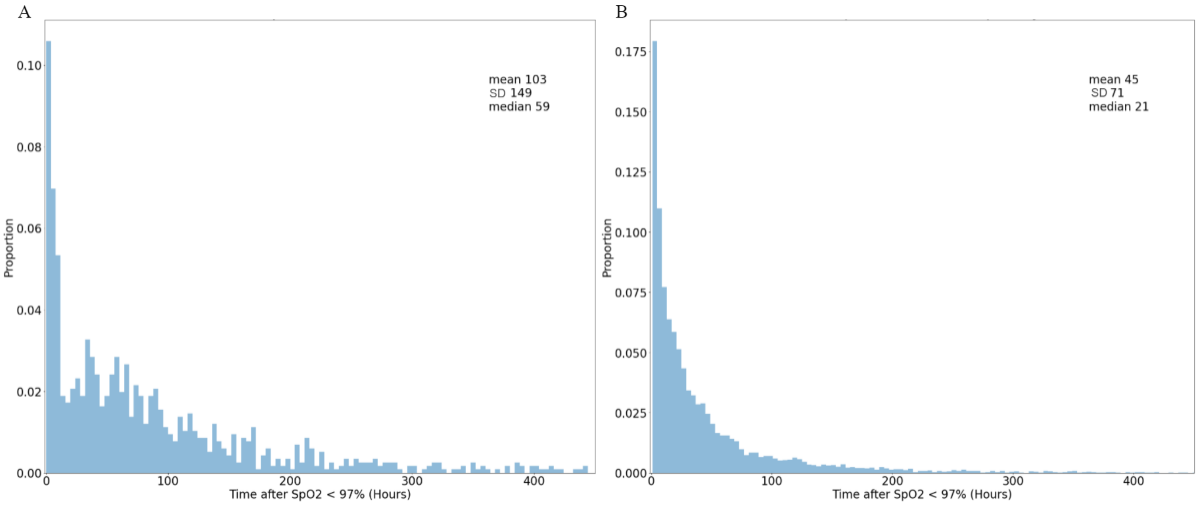
Prediction look-ahead times until (A) ARDS onset and (B) respiratory failure. The time until ARDS onset is the time after admission until any care provider adds the International Classification of Diseases code for ARDS into the electronic health record. The time until respiratory failure is the time after admission until the first measurement of an SpO_2_ level of <92% or a partial pressure of oxygen/fraction of inspired oxygen ratio of <300. These samples reflect the total data set. ARDS: acute respiratory distress syndrome; SpO_2_: peripheral oxygen saturation.

The median time interval from the prediction time until the onset of ARDS, which appeared as a diagnosis in patients’ EHRs, was 59 hours. The median time interval from the prediction time until the onset of respiratory failure, which appeared as a drop in _2_/FiO_2_ ratio of <300, was 21 hours. Histograms of the time intervals for the whole data set are shown in Figure 4, and those for the test set are shown in Figure S2 in Multimedia Appendix 1.

**Table 1:** Demographic information for the test population.

Demographic characteristics			Patients with ARDS^a^ (n=3383), n (%)		Patients without ARDS (n=74), n (%)
**Age (years)**					
	18-30	93 (2.7)		3 (4.1)	
	30-39	131 (3.9)		5 (6.8)	
	40-49	156 (4.6)		6 (8.1)	
	50-59	373 (11)		8 (10.8)	
	60-69	577 (17.1)		14 (18.9)	
	≥70	1886 (55.7)		34 (45.9)	
**Sex**					
	Male	1649 (48.7)		36 (48.6)	
	Female	1734 (51.3)		38 (51.4)	
**Race and ethnicity**					
	Non-Hispanic White	61 (1.8)		3 (4.1)	
	Non-Hispanic Black	23 (0.7)		1 (1.4)	
	Non-Hispanic Asian	1 (0)		0 (0)	
	Hispanic	3290 (97.3)		70 (94.6)	
	Non-Hispanic other	2 (0.1)		0 (0)	
	Unknown race or ethnicity	4 (0.1)		0 (0)	
**Comorbidities**					
	History of chronic pulmonary disease	126 (3.7)		9 (12.2)	
	History of cardiovascular disease	551 (16.3)		19 (25.7)	
	History of chronic heart failure	158 (4.7)		6 (8.1)	
	History of hypertension	242 (7.2)		14 (18.9)	
	History of diabetes	186 (5.5)		11 (14.9)	
	History of cancer	343 (10.1)		13 (17.6)	
	History of obesity	73 (2.2)		5 (6.8)	

^a^ARDS: acute respiratory distress syndrome.

The performance results of the initial teacher model and the semisupervised RNN model on the test data set are provided in Table 2. The best validation performance was achieved on cycle 3 of 4 during SSL training.

**Figure 5:**
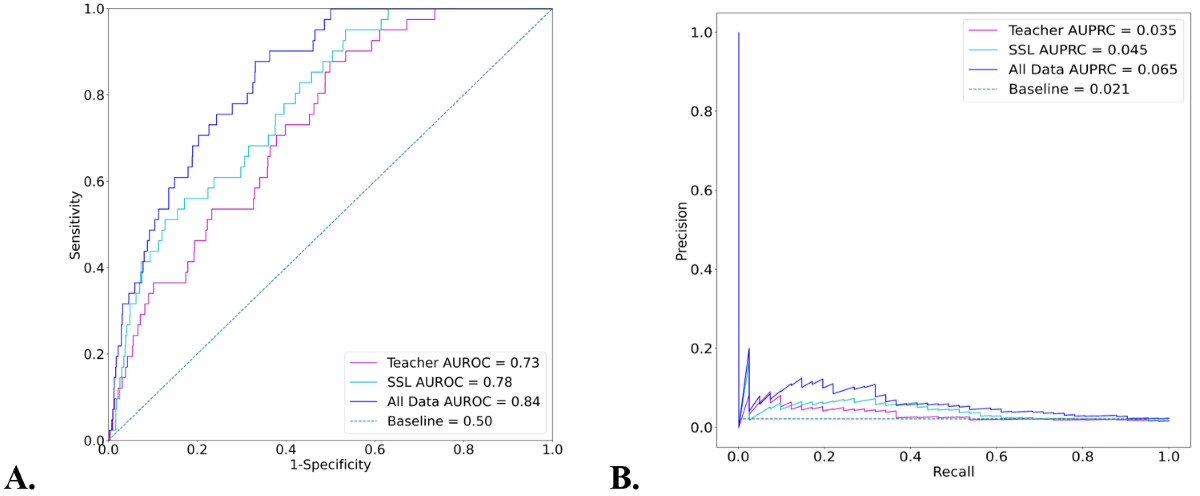
The (A) AUROCs and (B) AUPRCs for the predictions of acute respiratory distress syndrome onset made by the teacher, SSL, and all data models on the hold-out test set. AUPRC: area under the precision recall curve; AUROC: area under the receiver operating characteristic curve; SSL: semisupervised learning.

The results in Table 2 indicate that by using 16,173 unlabeled samples, we were able to use SSL to improve the model that was trained on the 6930 labeled samples. The amount of improvement was nontrivial compared to the performance that was possible when the model was trained on all data. The AUROCs and AUPRCs for the teacher, SSL, and all data models on the hold-out test set are presented in Figure 5. The same curves for auxiliary targets are provided in Figure S3 in Multimedia Appendix 1. Data on the subset of 489 patients with COVID-19 in the test set are shown in Figure S4 in Multimedia Appendix 1.

**Figure 6:**
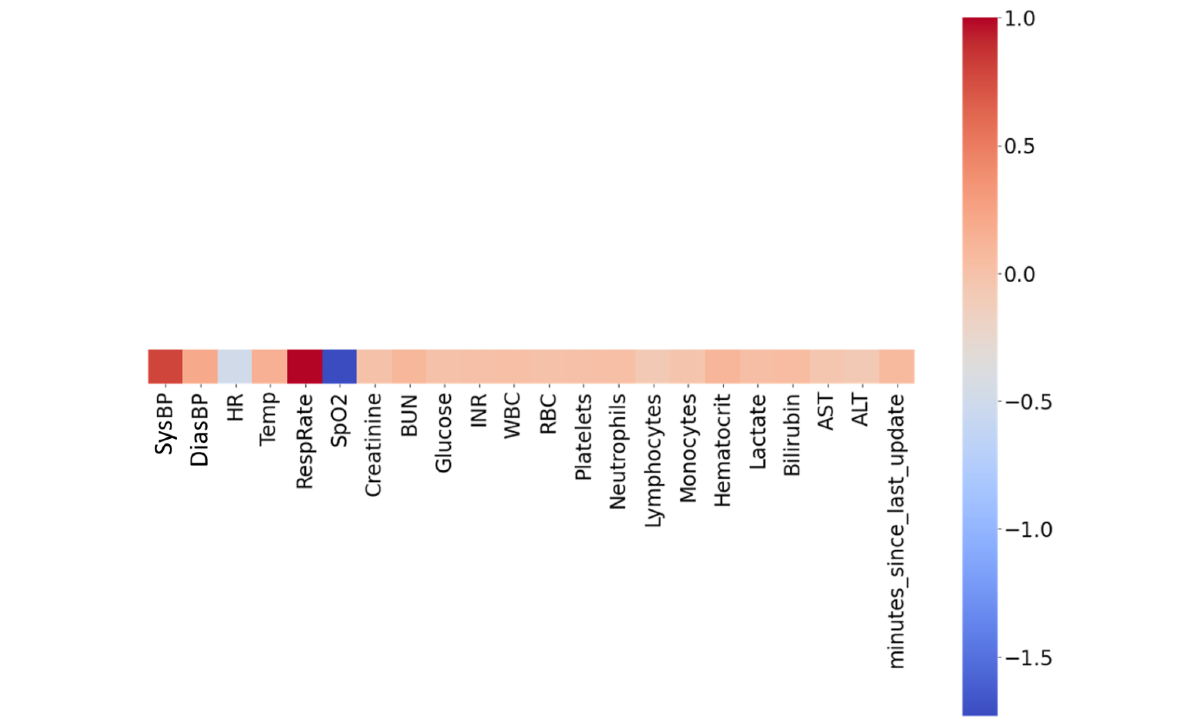
Feature inflection heat map. The mean z score of each time-varying feature at the time step with the greatest attention weight is shown. ALT: alanine transaminase; AST: aspartate transaminase; BUN: blood urea nitrogen; DiasBP: diastolic blood pressure; HR: heart rate; INR: international normalized ratio; RBC: red blood cell count; RespRate: respiratory rate; SpO_2_: peripheral oxygen saturation; SysBP: systolic blood pressure; Temp: temperature; WBC: white blood cell count.

The attention weights generated by the RNN were probed to visualize the signals that were attended to by the RNN. This method was used to implicitly describe the importance that was assigned to each feature by the model and provided some clues about model interpretability. For each patient in the test set, the time step with the greatest attention weight was extracted. This was the focus time step. The feature vector at this time step was interpreted as a *z* score for the subset of features that were measured during this particular time step. For example, a value of −0.5 in the heart rate dimension would denote that the heart rate is half an SD lower than the mean. For each time varying feature, we accumulated these directional inflections across all focus time steps and plotted a normalized heat map (Figure 6). Consistent with our intuition, the time steps with the greatest attention weights had large negative inflections in SpO_2_ level and large positive inflections in respiratory rate.

**Table 2:** Teacher and semisupervised learning model performance on test set.

Performance indicator	Initial teacher model	Semisupervised learning model	All data model
Area under the receiver operating characteristic curve	0.73	0.78	0.84
Area under the precision recall curve	0.035	0.045	0.065
Sensitivity	0.76	0.78	0.78
Specificity	0.55	0.61	0.72
Positive predictive value	0.020	0.023	0.033
Negative predictive value	0.995	0.996	0.996

## Discussion

### Principal Findings

We present a method of SSL for the early prediction of ARDS development. To address the challenges of poor label quality and limited data quantity, which make it difficult to predict ARDS development by using standard machine learning methods, we developed a method of SSL whereby confidently labeled data were assigned to a labeled data set and used for the testing, validation, and training of the RNN machine learning model. In the SSL scheme, the RNN model learned the latent representation of ARDS that was present in unlabeled data and expanded its own understanding of gold-standard labels. In doing so, the model established a relational link between a small set of commonly available clinical features and ARDS without needing to explicitly learn the Berlin definition of ARDS. To supplement the comparatively small labeled training data set, an unlabeled data set was pseudolabeled by an initial teacher RNN model. The pseudolabeled data were used for pretraining and were iteratively re-pseudolabeled by an evolving RNN-based machine learning model after the model was fine-tuned on the labeled training set. The SSL method was capable of accurately predicting ARDS development and was a considerable improvement over the baseline teacher model. Since the model was constructed by using a small subset of clinical features and outperformed a baseline model that was trained only on the small subset of labeled data, in practice, the model could be applied in settings where many clinical features are not available and settings where existing ARDS labels are incomplete or of low quality.

The paradigm described in this study differs from those in similar published machine learning studies because we apply an SSL methodology to the task of predicting the development of a severe respiratory condition (ie, a complication of COVID-19). In the case of other clinical conditions for which similar methodologies have been implemented (eg, predicting sepsis [26] and detecting microaneurysms and vascular lesions [27-29]), elements of the clinical definitions of such conditions can often be matched by using widely available EHR data. However, in the case of ARDS, measurements that can be used to create reliable gold-standard labels are not as widely available. This lack of data availability is detrimental to the supervised training of an ARDS prediction tool, as there may be many patient encounters that cannot be labeled as those involving ARDS and may in fact involve an episode of ARDS. If we had restricted ourselves to a supervised learning approach, which has been applied in the context of other clinical prediction tasks [30-32], our options for working with unlabeled data would have been limited. Alternatively, assigning these encounters a label of non-ARDS would have undermined the interpretation of performance metrics. We were therefore motivated to apply an SSL methodology to the task of ARDS prediction not only by the potential to improve upon our prior work [24] and to address new clinically relevant applications of machine learning, but also by the need to approach ARDS prediction in a fundamentally new way to address the practical challenges associated with a lack of reliably labeled retrospective data. Importantly, the prediction tool developed in this study can be used to accurately predict ARDS development without the requirement of radiographic data or subjective interpretation. Among general populations and COVID-19 populations in settings where radiographic information may not be available, the tool could be used to provide advance warning for ARDS onset and may allow for timely intervention. This would be particularly impactful for health care providers working in regions of lower socioeconomic status, where funding for advanced medical infrastructure and access to vaccines are limited, as these regions are known to have a higher incidence of burdens resulting from severe COVID-19 [33]. In addition, the SSL approach can leverage a small amount of costly labeled data (eg, during radiographic or manual adjudication by physicians for pseudolabeling a large amount of training data) to improve model performance.

There are several limitations to this study that lend themselves to opportunities for future work. To make the model applicable to a wide variety of clinical care settings, we simplified the model input features. Over the course of testing the SSL model, we also observed that model performance varied across clinical settings. It is possible that some hospitals may have collected features that were more important to making predictions or that features may have been collected more frequently in some hospitals than in others. In addition, most SSL methods involve some form of data augmentation in addition to pseudolabeling, and it remains an open question as to how to best perform data augmentation with clinical time-series data. In future work, we aim to determine if reinforcement learning is a suitable and mathematically rigorous methodology for the augmentation of clinical time-series data. Moreover, as we stressed earlier, predicting true ARDS development by using the Berlin definition requires radiology data. In the future, we would like to include radiology data in our model and compare the model presented in this study to the Berlin gold standard. On the other hand, our attention weight heat map (Figure 6) aims to provide insight about what signals were most attended to by the RNN. Although it provides useful data, information such as temporal change and the waveform of signals are lost in the heat map. Finally, model performance was only assessed based on retrospective patient data, and we were therefore unable to determine how the models might perform in prospective settings. Prospective validation is required to evaluate the impact of model predictions on patient outcomes.

### Conclusions

An SSL model was developed and externally validated for early ARDS prediction in both the general population and patients with COVID-19. Higher performance was achieved by the SSL model compared to that of the baseline teacher model for the general intensive care unit patient population. The semisupervised machine learning methodology allowed for early ARDS prediction in a manner that successfully mitigated the challenges that are commonly associated with a lack of reliably labeled data.

## Appendix

Multimedia Appendix 1 Supplementary material.

## Abbreviations

ARDS: acute respiratory distress syndrome
AUPRC: area under the precision recall curve
AUROC: area under the receiver operating characteristic curve
EHR: electronic health record
FiO_2_: fraction of inspired oxygen
GRU: gated recurrent unit
ICD: International Classification of Diseases
PaO_2_: partial pressure of oxygen
RNN: recurrent neural network
SpO_2_: peripheral oxygen saturation
SSL: semisupervised learning
