# Supplementary material for: Semisupervised Deep Learning Techniques for Predicting Acute Respiratory Distress Syndrome From Time-Series Clinical Data: Model Development and Validation Study
Source: JMIR Form Res. 2021 Sep 14;5(9):e28028. doi: 10.2196/28028 (PMC8447921; doi:10.2196/28028)
Supplement: Multimedia Appendix 1 [file formative_v5i9e28028_app1.docx]

**SUPPLEMENTARY MATERIAL**


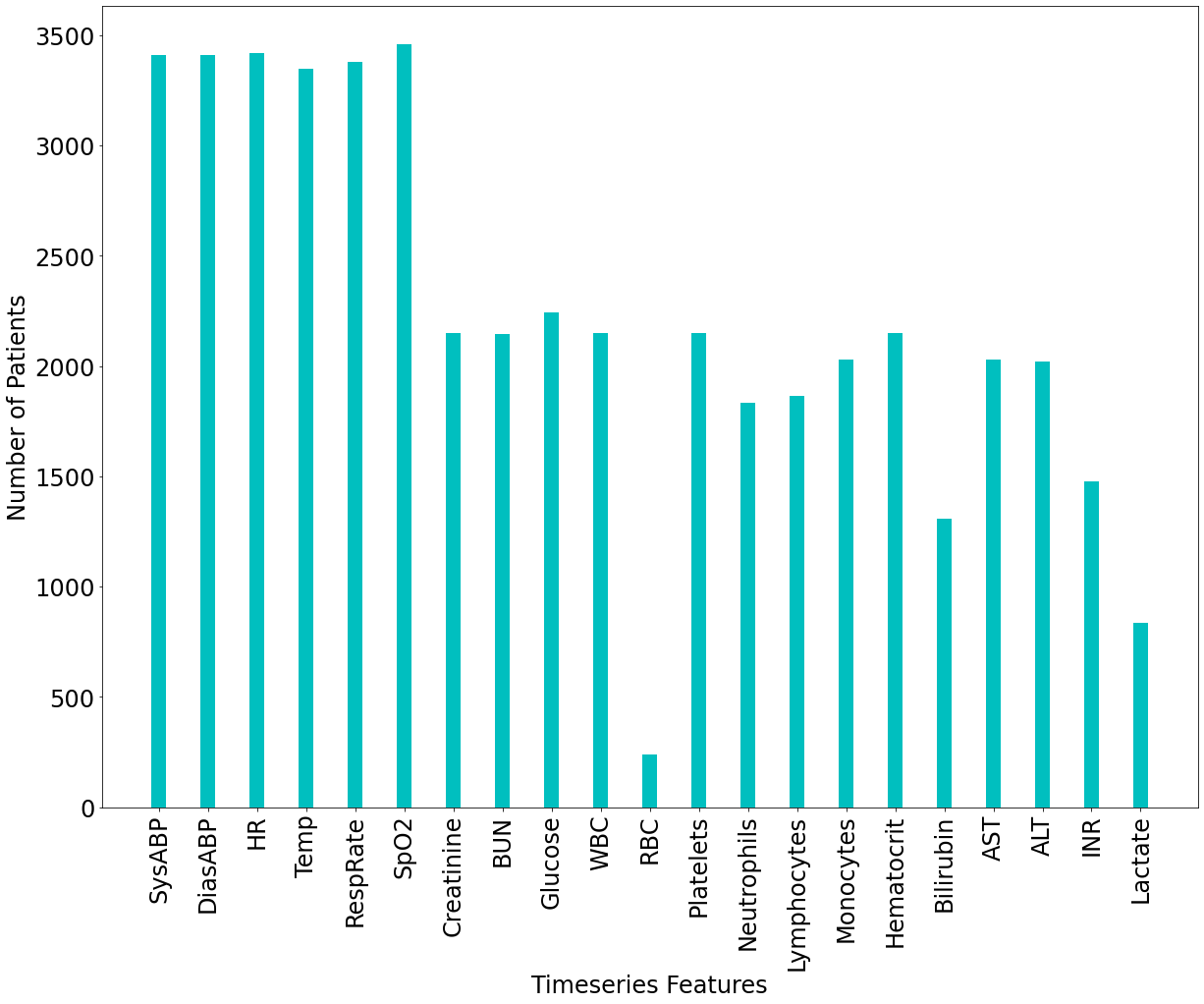


**Supplemental Figure 1.** Feature availability in the test set.

**Supplemental Table 1.** Feature Organization.

Raw Data

| Update time step | 0 | 1 | 2 |
| --- | --- | --- | --- |
| SysABP | 130 | NaN | 110 |
| SpO2 | 96 | NaN | 98 |
| Creatinine | NaN | 1.2 | NaN |
| Minutes since last update | NaN | 30 | 1 |

Normalized and Masked Data

| Update time step | 0 | 1 | 2 |
| --- | --- | --- | --- |
| SysABP | 0.1 | 0 | -0.1 |
| SpO2 | -0.2 | 0 | 0.01 |
| Creatinine | 0 | 0.001 | 0 |
| Minutes since last update | 0 | 0.5 | 0.001 |

| SysABP | 1 | 0 | 1 |
| --- | --- | --- | --- |
| SpO2 | 1 | 0 | 1 |
| Creatinine | 0 | 1 | 0 |
| Minutes since last update | 0 | 1 | 1 |


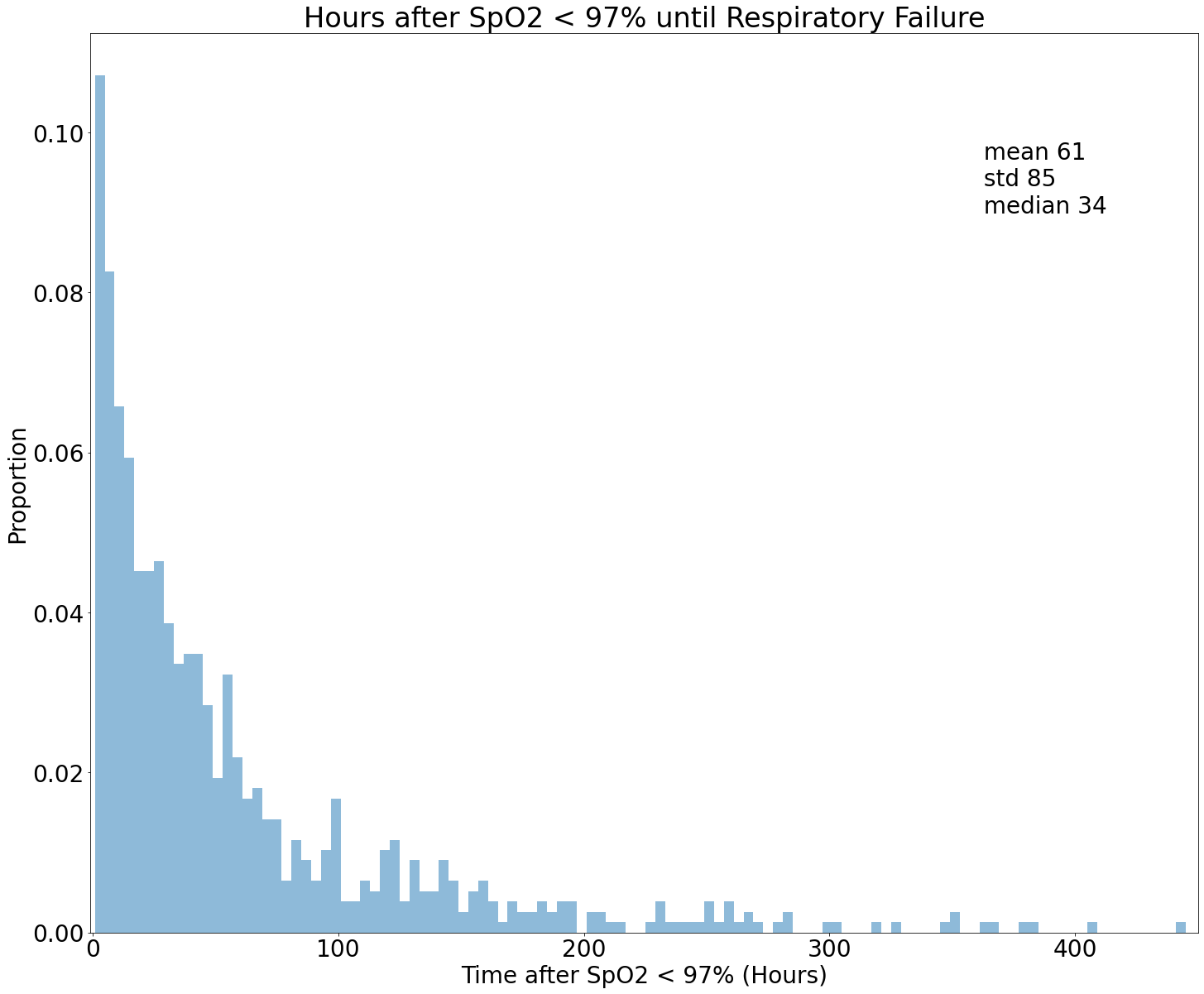

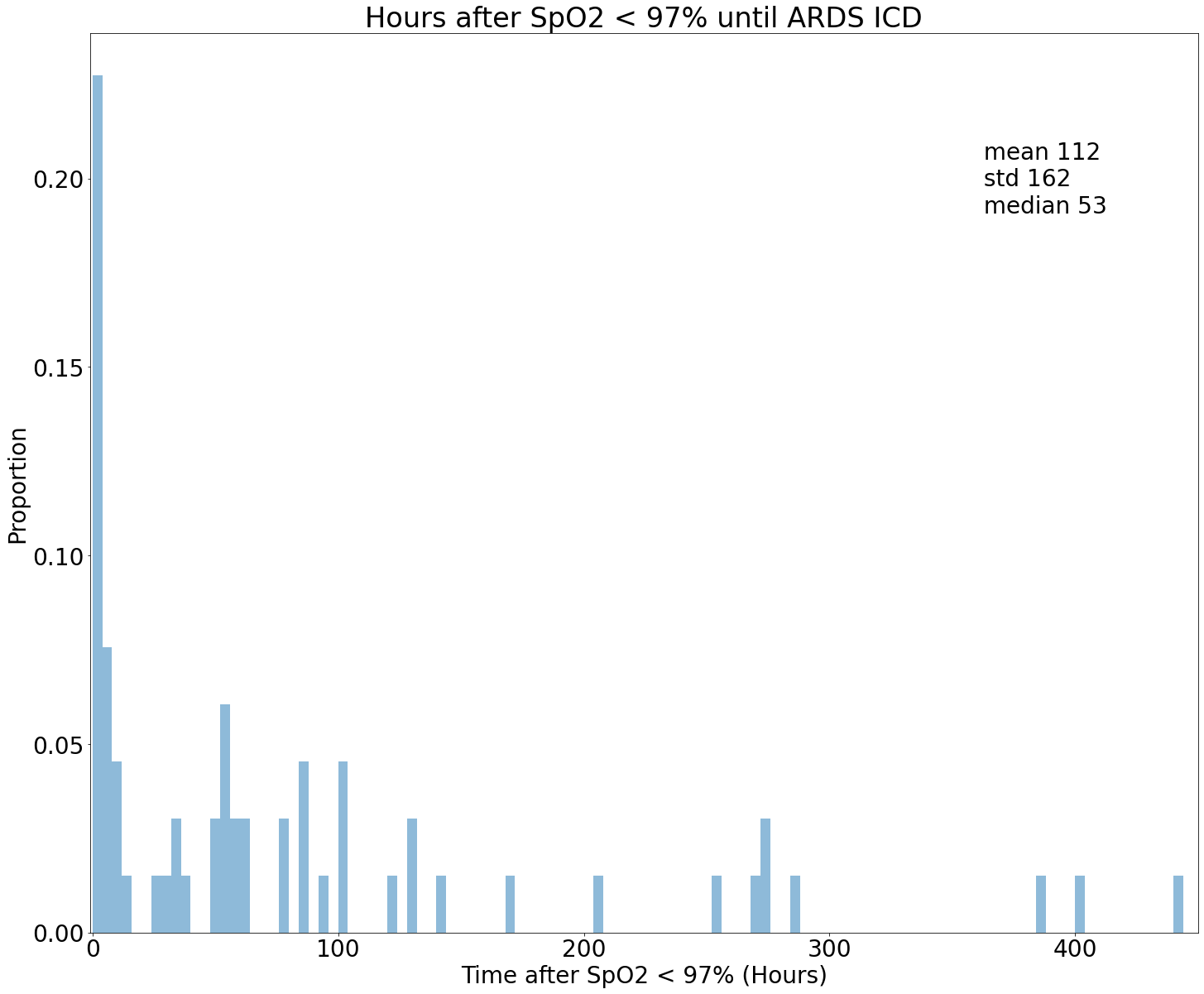


**Supplemental Figure 2.** Prediction Lookahead times until (Left) ARDS and (Right) Respiratory Failure . Time until ARDS is the time after admission until any care provider places the ICD code for ARDS into the EHR. Time until Respiratory Failure is the time after admission until the first measurement of an SpO2 < 92% or PF ratio < 300. These samples reflect the test dataset.


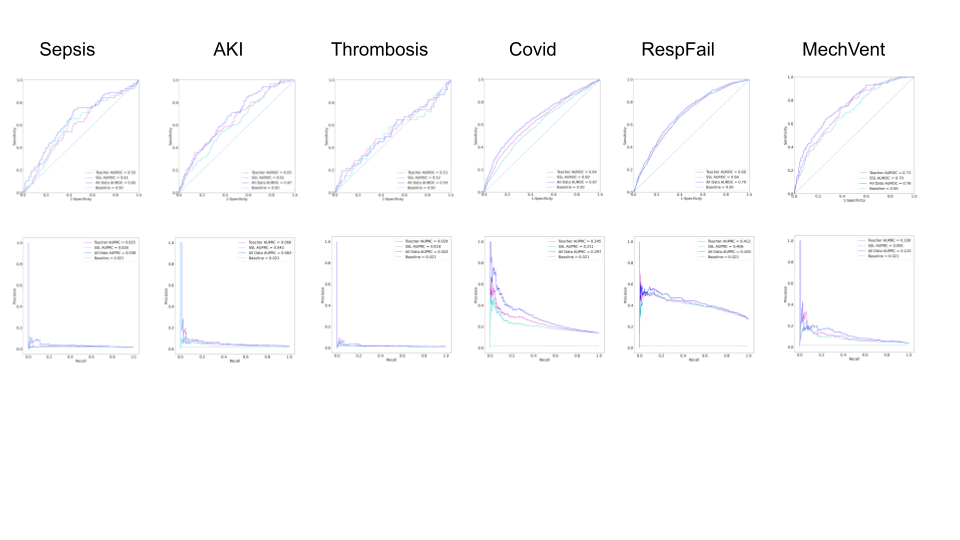


**Supplemental Figure 3.** Auxiliary Targets (Top) Area under the receiver operating characteristic (AUROC) and (Bottom) area under the precision recall curve (AUPRC) curves for prediction of ARDS onset for the Teacher, SSL and All Data models on the hold-out test set.


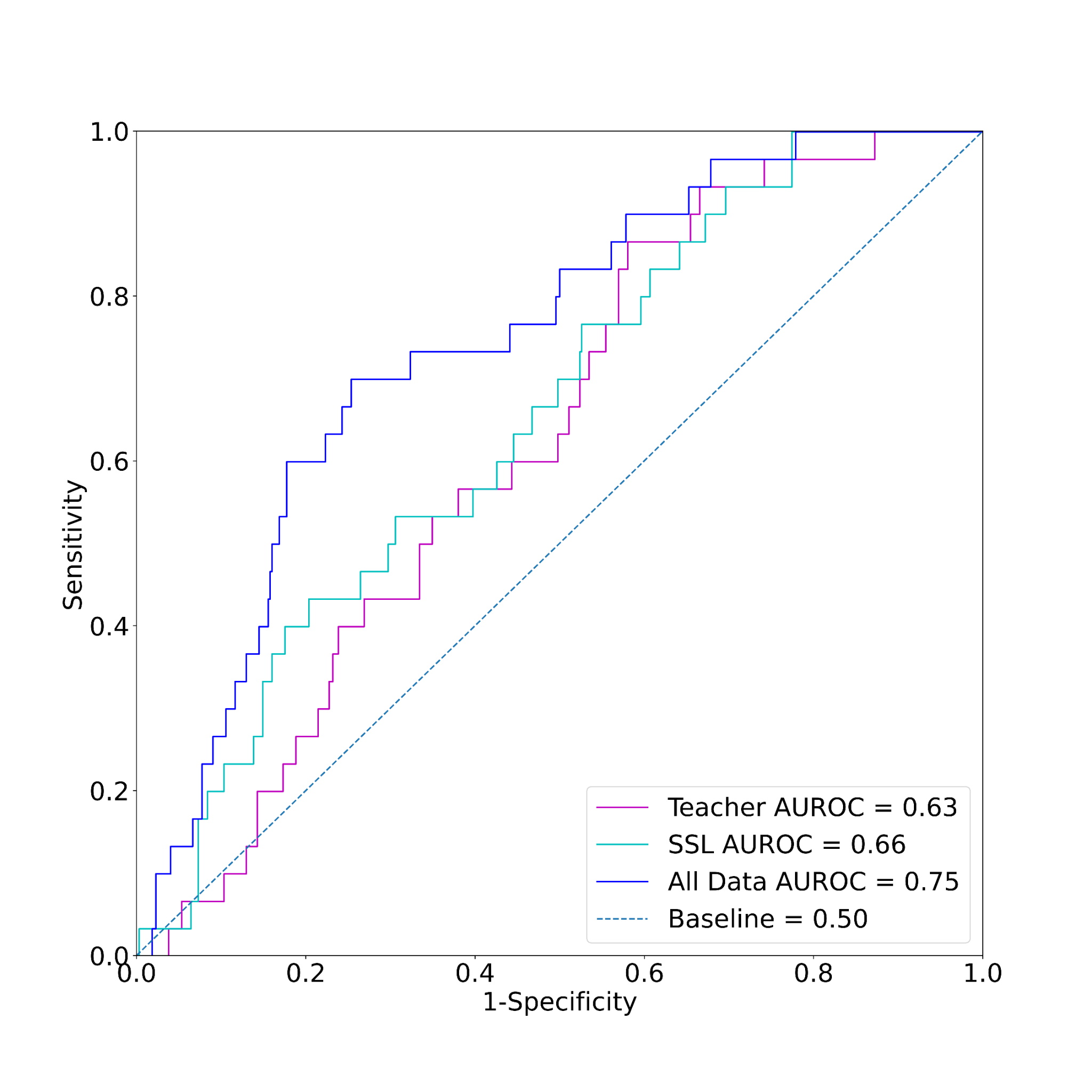

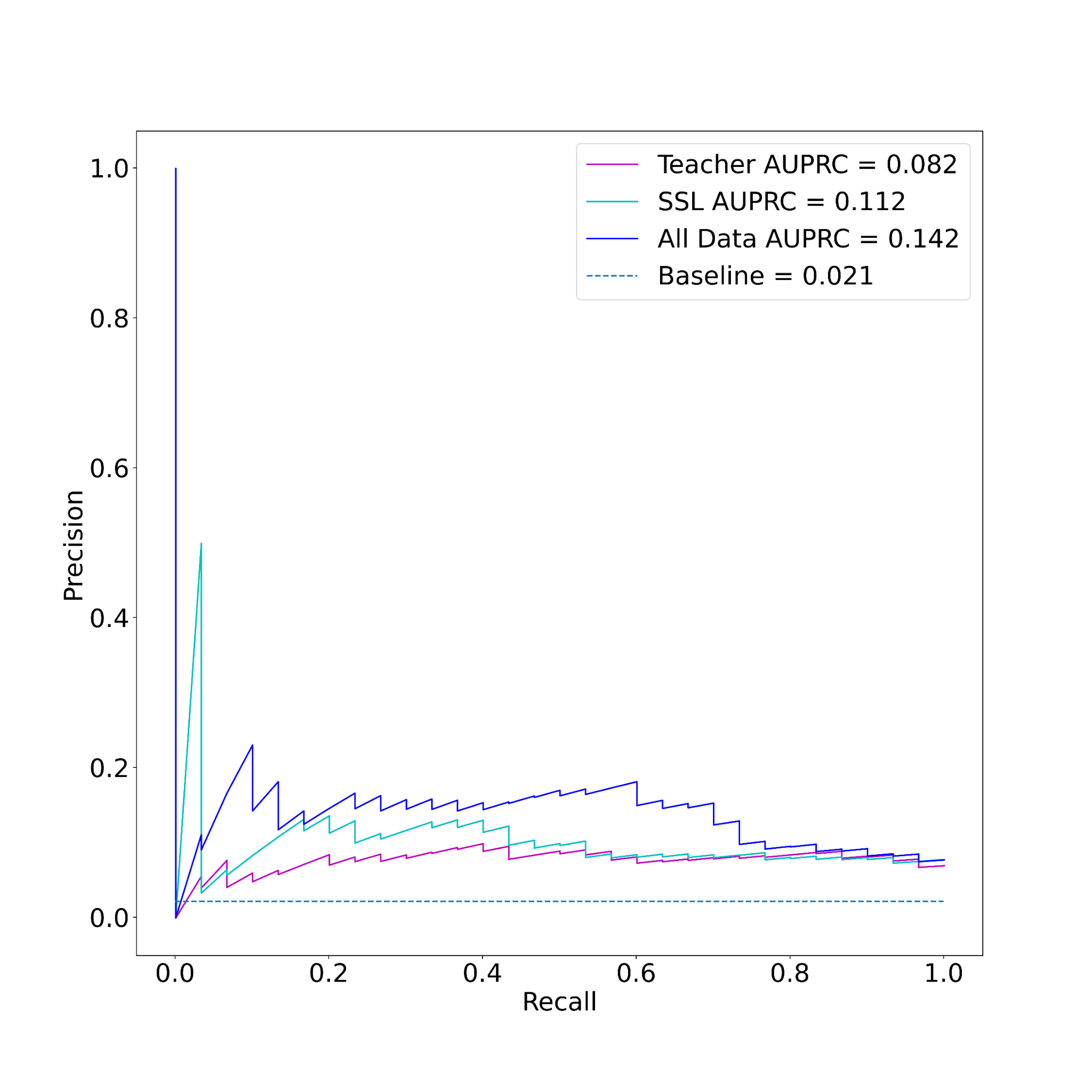


**Supplemental Figure 4.** Covid19 subpopulation (Left) Area under the receiver operating characteristic (AUROC) and (Right) area under the precision recall curve (AUPRC) curves for prediction of ARDS onset for the Teacher, SSL and All Data models on the hold-out test set.
